# Subpatent malaria in a low transmission African setting: a cross-sectional study using rapid diagnostic testing (RDT) and loop-mediated isothermal amplification (LAMP) from Zambezi region, Namibia

**DOI:** 10.1186/s12936-018-2626-5

**Published:** 2018-12-19

**Authors:** Patrick McCreesh, Davis Mumbengegwi, Kathryn Roberts, Munyaradzi Tambo, Jennifer Smith, Brooke Whittemore, Gerard Kelly, Caitlin Moe, Max Murphy, Mukosha Chisenga, Bryan Greenhouse, Henry Ntuku, Immo Kleinschmidt, Hugh Sturrock, Petrina Uusiku, Roland Gosling, Adam Bennett, Michelle S. Hsiang

**Affiliations:** 1 Malaria Elimination Initiative, Global Health Group, University of California, San Francisco (UCSF), San Francisco, CA USA; 2 Department of Pediatrics, University of Texas Southwestern Medical Center, Dallas, TX USA; 3 Department of Pediatrics, UCSF Benioff Children’s, San Francisco, CA USA; 4 Multidisciplinary Research Center, University of Namibia, Windhoek, Namibia; 5 Burnet Institute for Medical Research and Public Health, Melbourne, Australia; 6 Division of HIV, Infectious Diseases and Global Medicine, Department of Medicine, University of California, San Francisco, CA USA; 7 Department of Infectious Disease Epidemiology, London School of Hygiene and Tropical Medicine, London, UK; 8 Wits Research Institute for Malaria, University of Witwatersrands, Johannesburg, South Africa; 9 National Vectorborne Disease Control Programme, Namibia Ministry of Health and Social Services, Windhoek, Namibia

**Keywords:** Malaria, Malaria elimination, Subpatent, Submicroscopic, Subclinical, Asymptomatic, LAMP, Rapid diagnostic test, RDT, Namibia

## Abstract

**Background:**

Subpatent malaria infections, or low-density infections below the detection threshold of microscopy or standard rapid diagnostic testing (RDT), can perpetuate persistent transmission and, therefore, may be a barrier for countries like Namibia that are pursuing malaria elimination. This potential burden in Namibia has not been well characterized.

**Methods:**

Using a two-stage cluster sampling, cross-sectional design, subjects of all age were enrolled during the end of the 2015 malaria transmission season in Zambezi region, located in northeast Namibia. Malaria RDTs were performed with subsequent gold standard testing by loop-mediated isothermal amplification (LAMP) using dried blood spots. Infection prevalence was measured and the diagnostic accuracy of RDT calculated. Relationships between recent fever, demographics, epidemiological factors, and infection were assessed.

**Results:**

Prevalence of *Plasmodium falciparum* malaria infection was low: 0.8% (16/1919) by RDT and 2.2% (43/1919) by LAMP. All but one LAMP-positive infection was RDT-negative. Using LAMP as gold standard, the sensitivity and specificity of RDT were 2.3% and 99.2%, respectively. Compared to LAMP-negative infections, a higher portion LAMP-positive infections were associated with fever (45.2% vs. 30.4%, p = 0.04), though 55% of infections were not associated with fever. Agricultural occupations and cattle herding were significantly associated with LAMP-detectable infection (Adjusted ORs 5.02, 95% CI 1.77–14.23, and 11.82, 95% CI 1.06–131.81, respectively), while gender, travel, bed net use, and indoor residual spray coverage were not.

**Conclusions:**

This study presents results from the first large-scale malaria cross-sectional survey from Namibia using molecular testing to characterize subpatent infections. Findings suggest that fever history and standard RDTs are not useful to address this burden. Achievement of malaria elimination may require active case detection using more sensitive point-of-care diagnostics or presumptive treatment and targeted to high-risk groups.

**Electronic supplementary material:**

The online version of this article (10.1186/s12936-018-2626-5) contains supplementary material, which is available to authorized users.

## Background

Subpatent malaria infections, or low-density malaria infections below the detection threshold of standard rapid diagnostic tests (RDT) or microscopy, may be responsible for at least 20–50% of malaria transmission in pre-elimination and elimination settings [1–4]. As such, they are a key target for countries aiming to reduce or interrupt transmission [5]. These infections have generally been considered asymptomatic or minimally symptomatic, but there is also increasing data to suggest they are associated with adverse health effects including recurrent episodes of symptomatic parasitaemia, chronic anaemia, maternal and neonatal mortality, co-infection with invasive bacterial disease, and cognitive impairment [6].

A better understanding of the prevalence and characteristics of subpatent infections can inform the design of targeted strategies for malaria elimination [7–9]. Namibia is a low transmission country in southern Africa that has experienced a tremendous decline in reported malaria cases over the past decade due to the successful implementation of malaria control interventions. The country is now aiming to eliminate malaria [10], but the burden of subpatent infections has not been well-characterized [11, 12]. To address this gap, a large household-based cross-sectional survey was conducted in Zambezi region, Namibia. The study aimed to: (1) measure the prevalence of subpatent infection using loop-mediated isothermal amplification (LAMP), a molecular detection method, (2) measure the diagnostic accuracy of standard RDT using LAMP as gold standard, and (3) identify potential risk factors for subpatent malaria infection.

## Methods

### Study setting

**Fig. 1:**
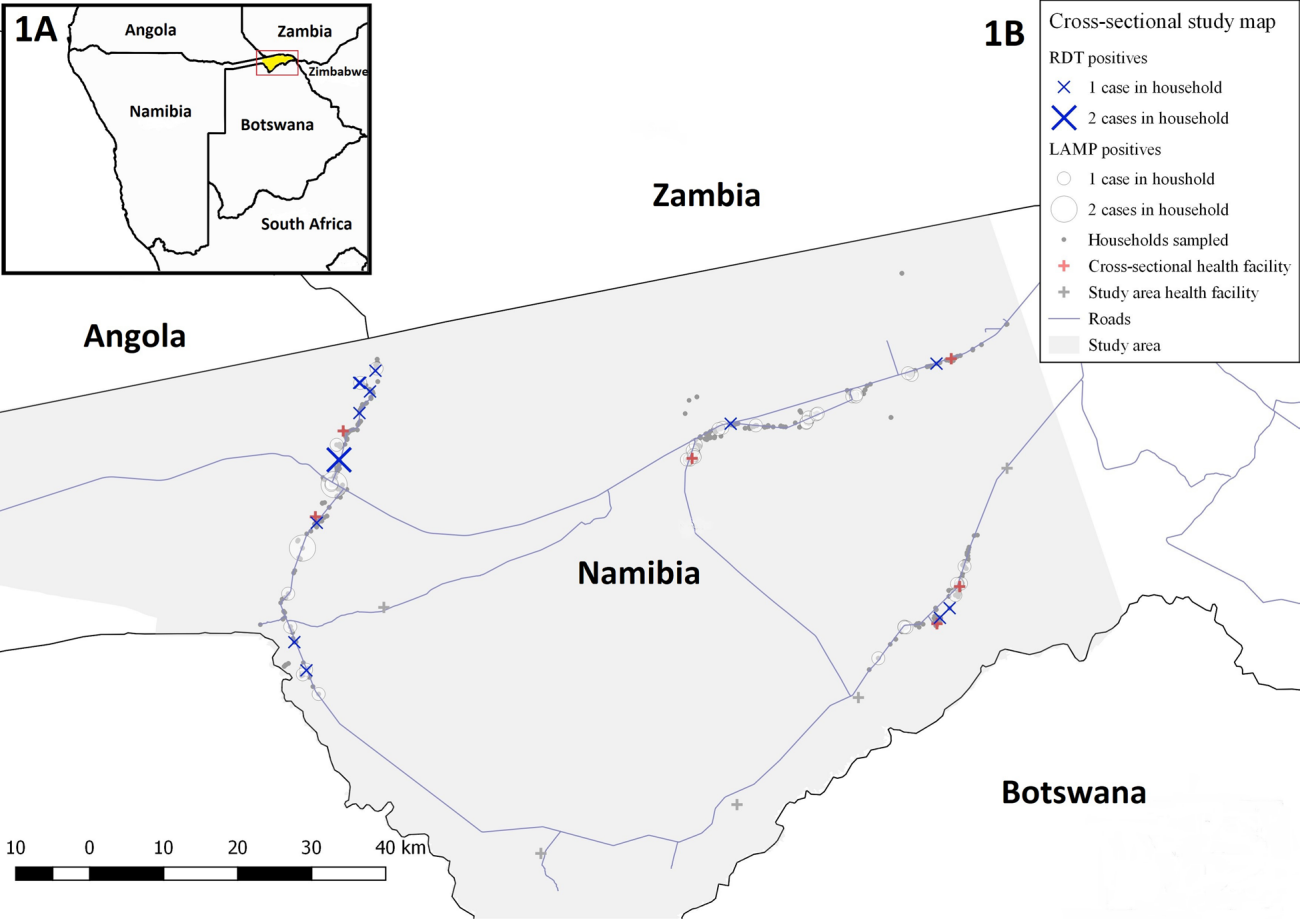
**a** Map of Zambezi region and study area, **b** spatial distribution of study participants and rapid diagnostic test (RDT) and loop-mediated isothermal amplification (LAMP) detected infections. *RDT* rapid diagnostic test, *DBS* dried blood spot, *LAMP* loop-mediated isothermal amplification

Zambezi region is located in the northern malarious area of Namibia, and shares borders with the higher malaria endemic countries of Angola and Zambia (Fig. 1a). It is a primarily rural setting with most of the population engaged in subsistence farming. From 2004 to 2015, the annual parasite incidence in Zambezi fell from over 600 to 17.1 cases per 1000 in 2014 [12, 13]. This decline in incidence occurred in the setting of improved implementation of indoor residual spraying (IRS), distribution and use of long-lasting insecticide-treated bed nets (ITNs), increased RDT use, and treatment of confirmed cases with artemisinin-based combination therapy. With high temperatures and significant seasonal rainfall, Zambezi region is the wettest region in Namibia. Following the wet season of October to May, the high transmission season typically begins in November, peaks between January and April, and then trails off in May. Reported malaria cases are almost all due to *Plasmodium falciparum* and the most common malaria vector species is *Anopheles arabiensis* [14].

### Study design and sample size

This cross-sectional survey was carried out at the end of the transmission season from April to June 2015 in the historically higher burden western part of Zambezi region (Fig. 1a), where based on the 2011 census data and projected growth rate, the estimated population size of the study area in 2015 was 35,381 [15]. In early 2015, the households in the study area were mapped and health facility catchment areas were defined, providing a household sampling frame for this survey. A two-stage, cluster sampling design was employed, where six of the eleven health facilities within the study area were randomly selected. Households from the selected health facility catchment areas were then randomly selected for inclusion in the survey.

The total target sample size was 2000 participants to detect a 5% prevalence based on LAMP with 80% study power and 5% significance level. These calculations assumed a 10% refusal rate, design effect of two (to account for correlations between clusters at the health facility level), annual parasite incidence (API) of at least 15/1000, 40% probability of clinical symptoms, 51% probability of seeing care at a health facility if febrile, 85% RDT diagnostic sensitivity for symptomatic infections, and 50% prevalence of annual incidence infections during the survey [16, 17]. Assuming an average of six individuals per household and that 10% of households would not be enrolled, 540 households was determined to be sufficient to reach the target sample size. However, 23% of households could not be enrolled due to absence or having moved away. Toward the end of the survey, an additional 144 households were randomly selected for recruitment.

### Participants

All individuals in selected households were eligible for inclusion in the survey if they were usual residents of the household or had slept in the household the night before. A complete line listing of all eligible individuals was generated at the first visit and subjects were excluded if they refused to participate, were too ill to participate, or were not present at any of three visits to each household.

### Data collection

Selected households were approached for written informed consent, which was conducted at the individual level and from a parent or guardian for minors less than 18 years of age. Additionally, minor assent was obtained for children > 12 years of age. Blood was collected by finger prick from all consenting participants for malaria testing by Carestart^™^ Malaria HRP2/pLDH (Pf/PAN) RDT (AccessBio, Somerset, NJ) and to generate a dried blood spot (DBS) using Whatmann^™^ 3 MM filter paper (GE Healthcare, Chicago, IL). Presence of clinical symptoms was assessed by self-report of fever in the prior 2 weeks. Infections associated with lack of self-reported fever were considered subclinical. Participants were interviewed in the local language SiLozi using a standardized tablet-based household questionnaire (administered to the household head or other adult representative) and individual questionnaire via Open Data Kit (ODK) software.

Household level data included the household location, socioeconomic status, household population, ITN ownership, as well as IRS coverage in the past 12 months. Questions assessing socioeconomic status included assessment of water source and household assets including electricity, radios, televisions, mobile phones, and refrigerators. The individual questionnaire assessed demographics, travel history, vector control coverage and use, history of malaria, and care-seeking behaviour associated with fever the past 2 weeks. All RDT positive individuals were considered to have uncomplicated malaria and received treatment in the field with artemether-lumefantrine according to national guidelines [18]. Subjects with severe malaria or illness not attributable to malaria were referred to the nearest health facility after assessment by study nurses. Up to two return visits were made to each household to maximize enrollment, with the goal to enroll at least 80% of residents within each household.

### Laboratory methods

DBS cards were transported to the University of Namibia Multi-Disciplinary Research Center laboratory in Windhoek. DNA was extracted using the Saponin/Chelex method previously described [19]. Using 15 µL of DNA extraction product, *Plasmodium*-specific testing was conducted using a commercial loop-mediated isothermal amplification kit (PAN-LAMP Loopamp detection kit, Eiken Chemical, Japan) in accordance with manufacturer’s instructions [20]. For quality assurance, all samples that were positive by RDT and/or PAN-LAMP and a random sampling of 10% of samples that were negative by RDT and LAMP underwent further testing by nested PCR (nPCR) targeting the cytochrome b gene, followed by species identification via *AluI* digest for nPCR positives [21]. Five microliter of DNA extraction product was used for nPCR. For any LAMP/nPCR discordant results, nPCR was repeated two additional times; nPCR was considered positive if at least one of the three results was positive. For LAMP and nPCR positive controls with parasite densities of at least 1 to 5 parasites/µL and 0.1 to 1 parasites/µL, respectively, were used.

### Data management and statistical analysis

Survey data were uploaded from tablet devices to a secure cloud-based sever at least weekly. Data were analysed using STATA (version 14.0; STATA Corp., College Station, TX, USA). Locations of cases were mapped using QGIS (version 2.16.2). Prevalence of malaria infection by RDT and LAMP were estimated and compared. Diagnostic accuracy of RDT was calculated using LAMP as gold standard. Based on the nPCR results of 10% of negative cases and all discordant LAMP and RDT samples, quality assurance of LAMP was assessed by calculating sensitivity and specificity of LAMP using nPCR as a gold standard. Demographic and epidemiological characteristics of participants were described. A housing quality variable was constructed by a principal component analysis based on type of wall, roof, window, and eaves. A socioeconomic status index was generated by principal component analysis based on six household assets ownership variables, wall type, roof type, and household water source.

Associations between covariates and LAMP-detectable malaria infection were explored using Chi squared and Fisher’s exact tests (Table 2). Epidemiological covariates with a *p* value of less than 0.10 in this bivariate analysis were included in multivariate logistic regression models, with gender, age, and socioeconomic status included a priori. Comparisons accounted for clustering at the highest level [22, 23] (by health facility catchment area) using robust standard errors. Reported fever in the prior 2 weeks was not included in the multivariate analysis due to it not being an epidemiological risk factor and its association with the outcome of infection. However, fever history was used for a stratified analysis of the diagnostic accuracy of RDT using LAMP as gold standard. Among the subset of individuals with fever history, the association between care-seeking behaviour and LAMP detectable malaria infection was also assessed separately. To investigate the distribution of malaria infection within the study area, spatial clusters of high malaria prevalence were identified using Kulldorff’s spatial scan statistic SaTScan™ v9.4 (SaTScan Boston, MA). Clustering was assessed using a spatial Bernoulli model (no other environmental covariates were included) with a maximum radius of 5 km and statistical significance tested by likelihood ratio, based on 999 Monte Carlo repetitions [24].

## Results

### Enrollment

**Fig. 2:**
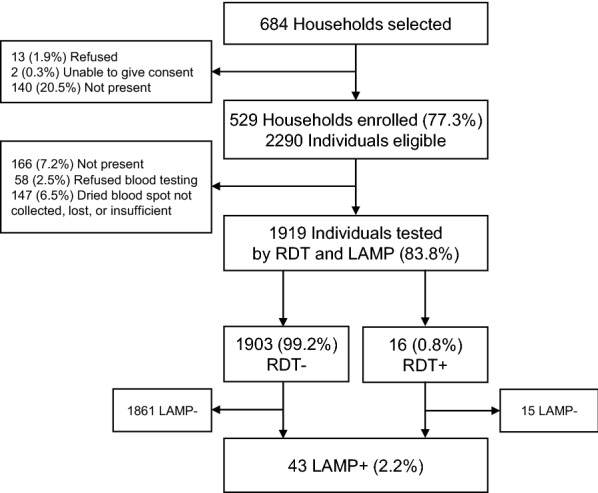
Enrollment and malaria testing results. *RDT* rapid diagnostic test, *LAMP* loop-mediated isothermal amplification

Of the 684 households sampled from the six catchment areas, 529 (77.3%) were enrolled (Fig. 2). Among 2290 eligible individuals in enrolled households, 1919 (83.8%) were tested by RDT and LAMP. The most common reason for lack of enrollment was that no household head was present after three visits by study teams. The most common reasons blood testing was not performed were absence (7.2%) and DBS not available (not collected, lost or insufficient blood) (6.5%).

### Study population characteristics

Study population characteristics are shown in Table 1. A slightly higher proportion of enrollees were female (55.0%) than male and the largest age group enrolled was children 15 years old or younger (47.0%). The most common occupation was agriculture representing 12.6% (241/1919) of all subjects, and 23.7% (241/1016) of the population over 15 years of age. Almost a quarter (23.1%) of the total adult population was unemployed. Fifteen percent of participants reported travel in the previous 1 to 8 weeks, with most travel being domestic (94.1%) rather than international (5.9%). Fever in the last 2 weeks was reported by 30.8% of participants. Overall, 33.1% of participants reported using an ITN the previous night and 59.6% of individuals lived in a household that received IRS in the past 12 months.

**Table 1:** Characteristics of study population, and association between clinical, demographic, and epidemiological factors and malaria infection

	Total (%) n = 1919	LAMP negative (%) n = 1876	LAMP positive (%) n = 42	p-value
Clinical				
Fever in the past 2 weeks				
No	1292 (69.2)	1269 (98.2)	23 (1.8)	0.04
Yes	574 (30.8)	555 (96.7	19 (3.3)	
Demographics				
Gender				
Female	1056 (55.0)	1033 (97.8)	23 (2.2)	0.84
Male	863 (45.0)	843 (97.7)	20 (2.3)	
Age (years)				
< 15	902 (47.0)	889 (98.6)	13 (1.4)	0.07
15–40	629 (32.8)	609 (96.8)	20 (3.2)	
> 40	387 (20.2)	378 (97.4)	10 (2.6)	
Occupation^a^				
Agricultural	241 (12.6)	229 (95.0)	12 (4.9)	0.001
Fishing	25 (1.3)	24 (96.0)	1 (4.0)	
Cattle herder	27 (1.4)	24 (88.9)	3 (11.1)	
Other manual labour	51 (2.7)	49 (96.1)	2 (3.9)	
Police officer/guard	16 (0.8)	16 (100)	0 (0)	
Office/commercial/professional	28 (1.5)	27 (96.4)	1 (3.6)	
Small market sales	27 (1.4)	27 (100)	0 (0)	
Unemployed/homemaker/retiree	443 (23.1)	438 (98.9)	5 (1.1)	
Student	146 (7.6)	142 (97.3)	4 (2.7)	
Other^b^	13 (0.7)	11 (84.6)	2 (15.4)	
≤ 15 years old	902 (47.0)	889 (98.6)	13 (1.4)	
Socio-economic status				
Lowest	368 (19.7)	360 (97.8)	8 (2.2)	0.99
Lower-middle	353 (18.9)	345 (97.7)	8 (2.3)	
Middle	380 (20.3)	372 (97.9)	8 (2.1)	
Upper-middle	384 (20.6)	376 (97.9)	8 (2.1)	
Highest	384 (20.6)	374 (97.4)	10 (2.6)	
Residence				
Health facility catchment area				
Chinchimane	179 (9.3)	174 (97.2)	5 (2.8)	0.83
Choi	308 (16.1)	300 (97.4)	8 (2.6)	
Kanono	226 (11.9)	221 (97.8)	5 (2.2)	
Kasheshe	340 (17.7)	331 (97.4)	9 (2.6)	
Sesheke	462 (24.1)	455 (98.5)	7 (1.5)	
Sibbinda	403 (21.0)	394 (97.8)	9 (2.2)	
Travel				
Travel in past 8 weeks				
No	1597 (85.5)	1560 (97.7)	37 (2.2)	0.17
Yes	270 (14.5)	263 (97.4)	7 (2.4)	
Travel categories				
No travel	1597 (85.5)	1562 (97.8)	35 (2.2)	0.76
Domestic travel	256 (13.7)	249 (97.3)	7 (2.1)	
International travel	16 (0.9)	16 (100)	0 (0)	
Housing				
Tertile of housing quality^c^				
Lowest	866 (46.4)	849 (98.0)	17 (2.0)	0.74
Middle	405 (21.7)	395 (97.5)	10 (2.5)	
Highest	594 (31.9)	579 (97.5)	15 (2.5)	
Individuals per household				
< 5	194 (10.6)	192 (99.0)	2 (1.0)	0.50
≥ 5 and < 10	1011 (55.1)	988 (97.7)	23 (2.3)	
≥ 10	629 (34.3)	613 (97.5)	16 (2.5)	
Vector control				
ITN ownership				
None	854 (45.6)	834 (97.7)	20 (2.3)	0.88
< 1 ITN per 2 people	647 (34.6)	634 (98.0)	13 (2.0)	
≥ 1 ITN per 2 people	371 (19.8)	362 (97.6)	9 (2.4)	
ITN use				
No	1250 (66.9)	1226 (98.1)	24 (1.9)	0.18
Yes	619 (33.1)	601 (97.1)	18 (2.9)	
Sprayed in past year				
No	723 (40.4)	710 (98.2)	13 (1.8)	0.36
Yes	1065 (59.6)	1039 (97.6)	26 (2.4)	

*LAMP* loop-mediated isothermal amplification, *RDT* rapid diagnostic test, *ITN* insecticide treated bed net, *IRS* indoor residual spraying

^a^Occupation was only assessed for participants over 15 years of age

^b^Herbalist, healer, bartender, land surveyor, soldier, headman

^c^Housing quality tertiles based on principle component analysis of wall type, roof type, eaves, and windows

### Prevalence of malaria infection

The overall prevalence of *Plasmodium* infection detected by RDT was 0.8%, 95% CI 0.4% to 1.2% (16/1919). By LAMP, prevalence was 2.2%, 95% CI 1.6% to 2.9% (43/1919) and in subsequent nPCR testing, all LAMP-positive infections were identified as *P. falciparum* mono-infection. Malaria prevalence by health facility catchment area ranged from 0 and 1.7% by RDT and 1.5 to 2.8% by LAMP. There were no statistically significant differences in malaria prevalence by health facility catchment area (χ^2^ = 1.80, p = 0.88).

Figure 1b shows the spatial distribution of study participants and RDT and LAMP detected infections, which reflects the population distribution along major roads. In the SatScan analysis, no statistically significant clusters of malaria infection were observed over the entire study area or within health facility catchment areas.

### Diagnostic accuracy

Compared to 2.2% infection prevalence by LAMP, prevalence of infection by RDT was only 0.8%. There was little concordance between RDT and LAMP positivity, with only one participant positive by both detection methods. Of seven symptomatic individuals who were RDT positive and LAMP negative, 2 (28%) reported treatment with artemether–lumefantrine within the past 2 weeks. Using LAMP as gold standard, the diagnostic accuracy of RDT was assessed in the total study population and in those with and without fever in the previous 2 weeks (Table 2). The sensitivities and positive predictive values were extremely low in all groups and specificities and negative predictive values high in all groups.

**Table 2:** Diagnostic accuracy of RDT in total study population and those with and without fever in the past 2 weeks, using LAMP as gold standard (n = 1919)

	Total (%, 95% CI)	Fever in past 2 weeks (%, 95% CI)	No fever in past 2 weeks (%, 95% CI)
Sensitivity	2.3 (0.06–13.8)	0 (0.0–20.9)	4.2 (0.2–23.1)
Specificity	99.2 (98.7–99.5)	98.7 (97.3–99.4)	99.4 (98.7–99.7)
Positive predictive value	6.3 (0.33–32.3)	0 (0–43.9)	11.1 (0.6–49.3)
Negative predictive value	97.8 (97.0–98.4)	96.6 (94.7–97.9)	98.3 (97.4–98.9)

Overall, the sensitivity and specificity of LAMP in the total study population using nPCR as the gold standard was 75.0% (95% CI 57.8–87.9) and specificity was 99.2% (95% CI 98.6–99.5), respectively. Among the samples that had repeat nPCR results, there were no discordant nPCR results. There was only one LAMP negative sample that was PCR positive. That all but one of the 15 RDT-positive LAMP-negative samples were confirmed negative by nPCR suggests that these samples were false RDT-positives (Additional file 1).

### Risk factor assessment

The associations between different malaria risk factors and LAMP detectable malaria infection are shown in Table 1. There was no evidence of association between LAMP detectable malaria infection and gender. In both the unadjusted and adjusted analysis (Table 3), occupational status was strongly associated with LAMP detectable infection. Compared to unemployed participants, and adjusting for gender, age, socioeconomic status, and clustering at the health facility level, cattle herders (AOR 11.82, 95% CI 1.06–131.81) and agricultural occupations (AOR 5.02, 95% CI 1.77–14.23) were significantly more likely to have a LAMP detectable malaria infection. There was no evidence of an association between age group or household socioeconomic status, and malaria infection.

**Table 3:** Logistic regression analysis for potential risk factors associated with LAMP detectable malaria infection (n = 1869)

Potential risk factor for malaria infection	OR (95% CI)	AOR* (95% CI)
Gender		
Female	Ref	
Male	1.07 (0.58–1.95)	0.99 (0.42–2.30)
Age (years)		
< 15	Ref	
15–40	2.25 (1.11–4.55)	1.35 (0.50–3.65)
> 40	1.81 (0.79–4.16)	0.79 (0.26–2.36)
Occupation		
Unemployed	Ref	
Student	2.47 (0.65–9.31)	2.12 (0.25–17.94)
Office/clerical/commercial/professional	3.24 (0.37–28.76)	3.44 (0.21–57.40)
Other manual labor	3.58 (0.68–18.92)	3.41 (0.11–107.45)
Fishing	3.65 (0.41–32.48)	3.51 (0.65–19.01)
Agricultural	4.55 (1.58–13.07)	5.02 (1.77–14.23)^a^
Cattle herder	14.21 (2.97–68.12)	11.82 (1.06–131.81)^b^
Other^c^	15.93 (2.78–91.25)	11.26 (1.02–124.69)
Socioeconomic status quintile		
Lowest	Ref	
Lower-middle	1.04 (0.39–2.81)	0.85 (0.39–1.89)
Middle	0.97 (0.36–2.61)	0.70 (0.22–2.21)
Upper-middle	0.96 (0.36–2.58)	0.71 (0.17–2.99)
Highest	1.20 (0.47–3.08)	0.85 (0.26–2.75)

*OR* odds ratio, *AOR* adjusted odds ratio

* Adjusted for gender, age, socioeconomic status, and clustering at the health facility level

^a^p-value = 0.01

^b^p-value = 0.04

^c^Herbalist, healer, bartender, land surveyor, soldier, headman

### Care-seeking behaviour

Of participants who reported fever in the previous 2 weeks (n = 571), only 51.8% (95% CI 48.4% to 55.3%) reported seeking care (Table 4). Compared to LAMP-negative subjects, LAMP-positive subjects were less likely to seek care (31.6% versus 52.5%), though the association did not reach statistical significance (p = 0.07). Among all individuals with recent fever and who sought care, almost all (91.9%) did so at a public health facility. The most common reason for seeking care was household proximity to a health facility (86.9%). The most common reasons for not seeking care for fever were lack of money for care or transportation (59.7%), and illness was not considered serious enough (15.2%).

**Table 4:** Characteristics of subjects with fever in prior 2 weeks and association between care-seeking behaviour and infection

Sought care	Total (%)	LAMP negative (%)	LAMP positive (%)	p-value
	n = 571	n = 552	n = 19	
No	275 (48.2)	262 (47.5)	13 (68.4)	0.07
Yes	296 (51.8)	290 (52.5)	6 (31.6)	

Care-seeking behaviour among subjects that sought care	Total (%)	LAMP negative (%)	LAMP positive (%)	p-value
	n = 296	n = 290	n = 6	
Treatment source				
Public	272 (91.9)	266 (91.7)	6 (100)	0.99
Other	24 (8.1)	24 (8.3)	0 (0)	
Days to seek treatment				
≤ 1 day	138 (46.6)	135 (46.6)	3 (50.0)	0.83
> 1 day and ≤ 1 week	141 (47.6)	138 (47.6)	3 (50.0)	
> 1 week	17 (5.7)	17 (5.9)	0 (0)	
Reasons for seeking treatment				
Close to home	246 (83.1)	240 (82.8)	6 (100)	1.00
Other	50 (16.9)	50 (17.2)	0 (0)	

Reasons for not seeking treatment among subjects that did not seek care	Total (%)	LAMP negative (%)	LAMP positive (%)	p-value
	n = 275	n = 262	n = 13	
Lacked money for care or transportation	157 (57.1)	152 (58.0)	5 (38.5)	0.18
Drugs not available	1 (0.4)	1 (0.4)	0 (0)	
Self-medication	13 (4.7)	11 (4.2)	2 (15.4)	
Long wait times at facility	3 (1.1)	3 (1.1)	0 (0)	
Distance/other transportation issue	17 (6.2)	17 (6.5)	0 (0)	
Illness not serious enough	40 (14.5)	36 (13.7)	4 (30.8)	
Other	44 (16.0)	42 (16.0)	2 (15.4)	

Care-seeking only assessed for those who reported fever in the past 2 weeks. Treatment source, days to seek treatment and reasons for seeking care only assessed for those who sought treatment. Reason for not seeking care only assessed for those who did not seek treatment

## Discussion

As a reservoir for persistent malaria transmission, subpatent malaria infections may pose a threat to countries like Namibia that are pursuing malaria elimination. Therefore, assessments of prevalence should not be limited to standard detection methods, such as microscopy and RDT. This study presents the results of the first large-scale survey in Namibia to using molecular methods to measure the prevalence of malaria infections. According to the survey LAMP results, prevalence of infection in the Zambezi region of northeastern Namibia was low at 2.2%, and almost half of prevalent infections were subclinical and almost none were detectable by the standard point-of-care diagnostic RDT. Among the demographic and epidemiologic risk factors assessed, agricultural and cattle herding occupations were significantly associated with LAMP positivity.

The low diagnostic accuracy of RDTs in this setting is consistent with other studies that have found limited sensitivity of RDTs to detect low density infections with less than 100 to 200 parasites/μL [25]. In a meta-analysis comparing diagnostics for the detection of asymptomatic *P. falciparum* infection in over 30,000 individuals, mainly from low transmission settings, the prevalence of infection by RDT was 41% of the prevalence detected by molecular testing [3]. Similarly, in this study, the 0.8% prevalence of infection by RDT represented 36.4% of the 2.2% prevalence by LAMP, and after excluding LAMP negatives (including 15 of the 16 RDT positives), sensitivity of RDT using LAMP as gold standard was 2.4% (1 of 43 LAMP positives). Apart from the limited sensitivity of RDT to low density infections, other potential explanations for the false negative RDT results were considered, such as limited sensitivity of the PAN/Pf RDT to detect non-falciparum infection [26]. However, all samples positive by genus-specific LAMP testing were positive by *P. falciparum* specific LAMP testing, and no non-falciparum infections were identified by PCR. Absence of HRP-2 antigen, the target for *P. falciparum* RDTs, has been reported in Africa and may be another contributing factor for false negative RDT results [27, 28]. Furthermore, 14 of the 16 RDT positives were LAMP or nPCR negative, potentially due to persistence of HRP-2 antigen which has been reported to remain in the bloodstream several weeks after successful treatment [29].

A key finding from this study was a higher risk of infection in individuals engaged in agricultural occupations and cattle herding. As it has been reported in other low transmission settings, outdoor occupations may increase exposure to mosquito bites [7, 30]. Furthermore, ITNs and IRS only provide protection from indoor biting. In low transmission settings, adult age has been frequently found to be associated with infection, presumably due to occupation and behavioural factors [7]. Results identified a trend of infection association with adult age, though the relationship was not statistically significant (Table 3). Male gender, bed net use, IRS coverage, lower socioeconomic status, and international travel, were not associated with infection, in contrast to results from a recent case–control study from Engela, in northern Namibia [31]. Travel may not appear to be associated with infection in the current survey because such a small proportion of the sample reported international travel (0.9% in Zambezi versus 5% in Engela). Also, because the survey was conducted in April and only assessed travel in the prior 8 weeks, it did not capture travel details from the holiday season when travel peaks. Future work to better understand imported malaria should obtain more detailed travel history [32]. Finally, as geographical clustering of infections was not observed, there was no clear spatial risk associated with malaria infection. This could be due to the small number of cases observed. Also, the study was not powered or designed to examine spatial clustering.

To examine coverage or availability of health care services, health-seeking behaviour was assessed among subjects who reported fever in the prior 2 weeks. There were no differences between LAMP positive and negative subjects in terms of care-seeking at a private versus public facility, time between onset of symptoms and care-seeking, or reasons for seeking or not seeking care. However, there was a trend in the association between LAMP positivity and not seeking care. Improved healthcare access could decrease the number of people in communities with malaria symptoms who do not seek care and consequently increase the likelihood of transmitting infections to mosquitos. However, if these infections were subpatent at time of presentation, there may be indication for more sensitive diagnostics in the clinical setting. Even with improved malaria care-seeking and improved point-of-care diagnostics in the clinical setting, there remains the challenge of subclinical infections, which may require a community-based screening and treatment approach using more sensitive diagnostics, or presumptive treatment [33].

There were some limitations to this study. First, cross-sectional designs are suboptimal for identification of risk factors in low transmission settings due to the low number of cases and challenges in determining causality in the relationships between risk factors and prevalent infections. Indeed, the malaria incidence in 2015 was historically low, largely due to a drought in southern Africa. Another drawback was that the classification of subclinical infection was based on self-reported history of fever in the prior 2 weeks, a subjective assessment which may not accurately reflect symptomatology. Further, chronic subpatent infection can persist for months and the fever assessment of just 2 weeks was limited [34]. Cohort studies with regular measurement of temperature and more detailed assessment of symptoms would be a stronger study design, but impractical in this low transmission setting due to the low prevalence of infection. Finally, very low-density infections may have been missed because high blood volumes were not collected and an ultrasensitive molecular detection method was not used [35]. There is a growing body of evidence suggesting near equivalence of LAMP to nPCR [20, 36–38] and the use of a higher volume of template DNA for LAMP compared to nPCR likely helped to improve the sensitivity of LAMP. However, to better elucidate the extent of low-density infection and the numerical distribution of parasite density, future studies could utilize more sensitive detection methods.

This study is the first large-scale malaria cross-sectional survey in Namibia using molecular testing and highlighted the importance of subpatent infections in this low transmission setting [11]. Fever history and standard RDTs do not appear useful to address this burden. Achievement of malaria elimination may require active case detection using more sensitive point-of-care diagnostics or presumptive treatment (e.g. mass drug administration). Agricultural occupations and cattle herding were associated with a higher risk of infection and achievement of malaria elimination will likely require intervention strategies that target this high-risk population.

## Additional file

**Additional file 1.** RDT and LAMP results used to calculate diagnostic accuracy of RDT using LAMP as gold standard (n = 1919).

## Abbreviations

RDT: rapid diagnostic tests
LAMP: loop mediated isothermal amplification
HRP2: histidine-rich protein 2
ITN: insecticide-treated bed net
IRS: indoor-residual spraying
nPCR: nested PCR
ODK: Open Data Kit
DBS: dried blood spot
