# Supplementary material for: Subpatent malaria in a low transmission African setting: a cross-sectional study using rapid diagnostic testing (RDT) and loop-mediated isothermal amplification (LAMP) from Zambezi region, Namibia
Source: Malar J. 2018 Dec 19;17:480. doi: 10.1186/s12936-018-2626-5 (PMC6299963; doi:10.1186/s12936-018-2626-5)
Supplement: Supplementary file 1 — Additional file 1. RDT and LAMP results used to calculate diagnostic accuracy of RDT using LAMP as gold standard (n = 1919). [file 12936_2018_2626_MOESM1_ESM.docx]

**Additional file 1.** RDT and LAMP results used to calculate diagnostic accuracy of RDT using LAMP as gold standard (n=1919)

|  | | LAMP Result | |  |
| --- | --- | --- | --- | --- |
|  |  | Positive | Negative | Total |
| RDT Result | Positive | 1 | 15 | 16 |
|  | Negative | 42 | 1861 | 1903 |
|  | Total | 43 | 1876 | 1919 |

RDT: Rapid Diagnostic test; LAMP: Loop-mediated isothermal amplification

*For quality assurance, nested PCR (nPCR) was performed on all samples positive by RDT and/or LAMP and 10% of samples negative by RDT and LAMP. All but one LAMP-negative sample was nPCR-negative (n=222). Of LAMP-positive samples, 27 of 43 were nPCR-positive, including the 1 RDT-positive/LAMP-positive sample.
